# Full-time bracing versus hybrid bracing in adolescent idiopathic scoliosis: study protocol for a randomised controlled trial

**DOI:** 10.1186/s13063-026-09787-0

**Published:** 2026-05-19

**Authors:** Adam Yiu Chung Lau, Ho Man Kee, Kenneth Guangpu Yang, Eric Cheuk Kin Kwan, Alec Lik Hang Hung, Wayne Yuk Wai Lee, Man Sang Wong, Jack Chun Yiu Cheng, Tsz Ping Lam

**Affiliations:** 1 SH Ho Scoliosis Research Laboratory, Joint Scoliosis Research Centre of The Chinese University of Hong Kong and Nanjing University, Department of Orthopaedics and Traumatology, The Chinese University of Hong Kong, Hong Kong, SAR China; 2 Prosthetics and Orthotics Department, Prince of Wales Hospital, Hong Kong, SAR China; 3 Department of Biomedical Engineering, The Hong Kong Polytechnic University, Hong Kong, SAR China

**Keywords:** Adolescent idiopathic scoliosis, Bracing, In-brace correction

## Abstract

**Background:**

Adolescent idiopathic scoliosis (AIS) is a complex three-dimensional spinal deformity affecting 2–4% of adolescents. During the peripubertal stage, untreated AIS may progress rapidly and lead to adverse outcomes such as back pain and poor body image. Full-time bracing, such as the underarm brace (UAB), is indicated for skeletally immature patients with a Cobb angle greater than 20°. Nighttime lateral bending braces (LBB) aim to achieve overcorrection by positioning the patient in a supine bending position opposite to the curve convexity. Despite bracing, 20–30% of patients experience progression requiring surgery. In-brace correction (IBC) is a key determinant of bracing effectiveness. To enhance IBC, we propose a Hybrid Bracing Protocol (HyBP) combining daytime UAB and nighttime LBB.

**Objective:**

The objective of this trial is to determine whether the Hybrid Bracing Protocol achieves greater in-brace correction (IBC) compared with the conventional bracing at 3 months and 18 months after brace initiation. This 18-month study represents the first phase, followed by a second phase assessing curve outcomes at brace completion and 2 years post-bracing.

**Methods:**

This is a prospective, randomised, parallel-group trial comparing the Hybrid Bracing Protocol (HyBP) with conventional bracing for enhancing in-brace correction (IBC). Skeletally immature female patients with adolescent idiopathic scoliosis and a single thoracolumbar or lumbar curve newly prescribed for bracing will be randomly allocated to either the HyBP group or conventional brace group (CB group).

**Discussion:**

To our knowledge, this will be the randomised trial to evaluate the potential synergistic effect of combining two different bracing designs within a hybrid protocol. If effective, this approach may improve bracing outcome, reduce curve progression, and decrease the need for surgical intervention.

**Trial registration:**

ClinicalTrials.gov NCT07045337. Registered on July 9, 2025.

**Supplementary Information:**

The online version contains supplementary material available at 10.1186/s13063-026-09787-0.

## Background

Adolescent idiopathic scoliosis is a complex three-dimensional spinal deformity with a prevalence of 2–4%. It commonly occurs in adolescents between 10 and 16 years old with a female to male ratio of 7:1 for curves greater than 30 degrees [1]. If left untreated, AIS can progress and be associated with serious health problems including back degeneration, cardiopulmonary compromises, negative body images, and psychosocial disorders arising out of grossly deformed torso [2].

In general, AIS patients with Cobb angle < 20° are closely monitored at regular intervals. For skeletally immature patients having Cobb angle greater than 20 to 25°, bracing is indicated [3]. Bracing can be defined as the application of external corrective forces to the trunk with the goals of halting curve progression in AIS [4]. Rigid braces such as the underarm brace are frequently prescribed in clinical settings. Apart from the active mechanisms with muscle control to shift the trunk away from the pressure areas, a passive mechanism with 3-point external forces applied by the brace pads could be operating and account for the orthotic control of curve progression in AIS [5].

Weinstein et al. [6] conducted the BrAIST study which was a prospective randomised controlled trial on full-time bracing and analysed both the randomised and preference cohorts and concluded that bracing could be useful in a proportion of cases for controlling curves from progression to the threshold for surgery, some cases do progress even with bracing. Surgery is considered when curves become severe (Cobb angle > 50°). It is typically in form of spinal fusion resulting in permanent loss of motion of the fused spinal segment. Although surgery can effectively alleviate and stabilise severe curves, it is a major and invasive procedure associated with serious complications including massive blood loss, wound infections, implant failures, spinal cord injuries, and even mortality.

In view of the invasive nature of surgical procedures and the significant morbidities with severe scoliosis, it is important to maximise the effectiveness of bracing treatment for AIS to avoid the need for surgery. The effectiveness of bracing hinges on various factors, one of which is the in-brace curve correction, i.e. in-brace correction (IBC) [7]. IBC is defined as the percentage of Cobb angle reduction during an X-ray with the brace fitted on the patient.

$$\text{In-brace Correction (IBC)}=(([\text{Pre-brace Cobb}]-[\text{In-brace Cobb}]/[\text{Pre-brace Cobb}])\times 100\%$$

Bogaart’s review showed strong evidence that a lack of initial IBC is associated with treatment failure [8]. Karavidas in another systematic review concluded that initial IBC seemed to be the most important predictive factor for successful treatment outcome [9]. With strong evidence from previous observational studies that IBC is a key determinant of treatment outcome for bracing, it is logical to explore ways of maximising treatment effectiveness of bracing through enhancement of IBC. With our early pilot results, we propose that a lateral bending brace (LBB) can stretch the concave side of the curve, enhancing the spinal flexibility and resulting in an improved IBC within a daytime underarm brace abbreviated as UAB, and we hypothesise that the HyBP has better treatment effect for AIS in terms of IBC and control of curve progression when compared with the conventional full-time UAB.

Objectives of this study:

1. The primary objective is to determine if the Hybrid Bracing Protocol (HyBP) combining the use of daytime underarm brace (UAB) and a nighttime lateral bending brace has better treatment effect in terms of in-brace correction (IBC) when compared with the conventional full-time UAB.
2. The secondary objectives are:
  - To determine if the HyBP has better treatment outcomes in terms of control of curve progression when compared with the conventional full-time UAB for those who have completed bracing treatment or require surgery by the end of this 3-year study proposal
  - To determine if the HyBP has better outcomes when compared with the conventional full-time UAB in terms of quality of life as assessed by SRS-22r, Spinal Appearance Questionnaire, and the Brace Questionnaire

## Methods

### Trial design

**Fig. 1:**
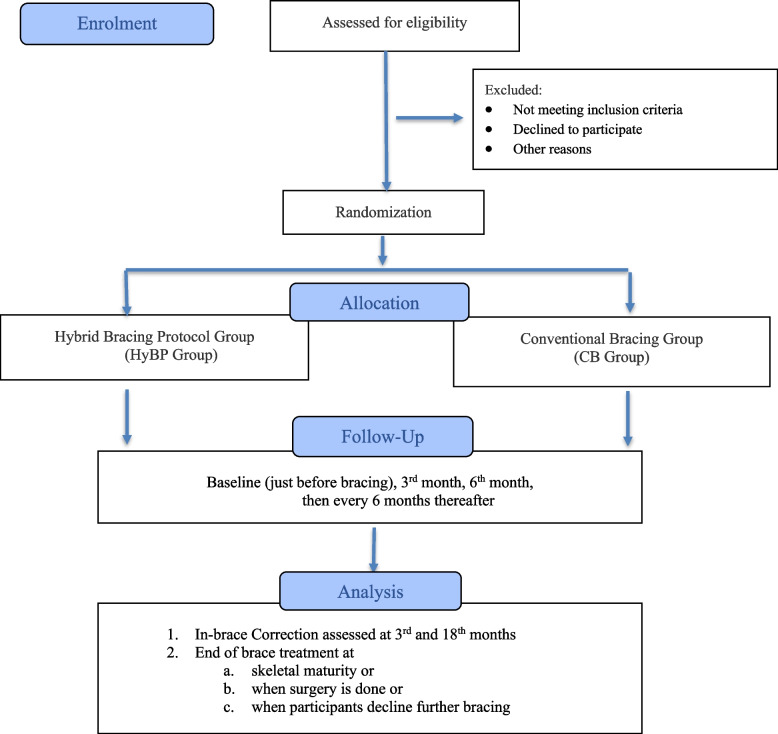
CONSORT flow diagram

**Fig. 2:**
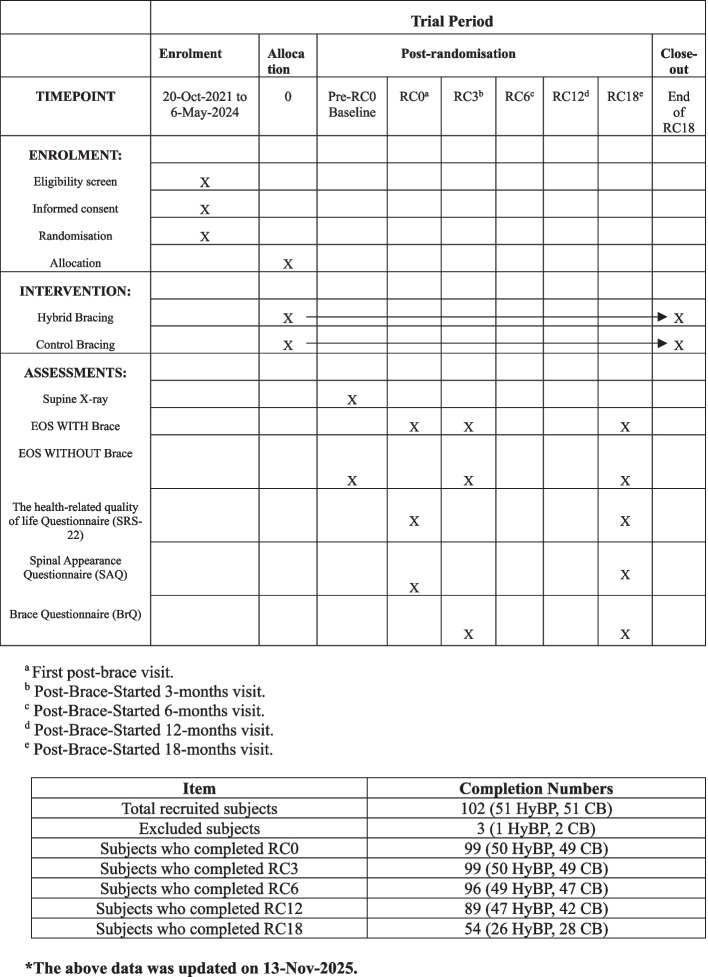
SPIRIT figure

A prospective single-centre, parallel-group, non-blinded, randomised-controlled trial will be conducted (Fig. 1). The trial is registered with ClinicalTrials.gov NCT07045337. SPIRIT reporting guideline is used [10] and ethical approval is obtained from the Clinical Research Ethics Committee of the University and Hospital before commencement (CREC no. 2021.114-T). The first phase of study is supported by the General Research Fund (No. 14119721, Research Grants Council, Hong Kong), to be followed by the second phase of the project when all subjects of the entire cohort will be assessed on curve outcome at time of completion of bracing and at 2 years post-bracing (Fig. 2).

### Participant recruitment

Eligible skeletally immature female AIS subjects newly prescribed with bracing will be enrolled from a tertiary referral scoliosis outpatient clinic. An initial interview will be conducted with the subject and her legal guardians on details of the aims, the protocol, and the timeframe of the study. Questions from the participants will be answered up to their satisfaction. Written informed consent will be obtained from all participants and their legal guardians before enrolment.

#### Inclusion criteria

1. Females with AIS diagnosed after detailed clinical and radiological evaluation by an experienced orthopaedic surgeon and
2. Having a single lumbar or thoracolumbar curve with Cobb angle between 20 and 40° and
3. Age 10 years and older when bracing is prescribed and
4. Risser 0 to 2 and
5. Either pre-menarchal or less than 1 year post-menarchal and
6. With no prior treatment for scoliosis

#### Exclusion criteria

1. Presenting with associated musculoskeletal, neurological, or other conditions possibly responsible for the curvature
2. With previous surgical or orthotic treatment or
3. Physical or mental disability that prevents patients from complying with the bracing protocol

### Sample size estimation

**Table:** 

	HyBP group (mean ± SD)	Conventional brace group (mean ± SD)	*p* value (Mann-Whitney test)
Number of subjects	8	8	
Curve type	All had single thoracolumbar or lumbar curve		
Age (years)	13.0 ± 0.8	11.7 ± 0.8	0.012
Risser	3.1 ± 1.0	1.5 ± 1.9	0.072
Cobb angle	25.5 ± 4.7	26.8 ± 5.6	0.560
In-brace correction	42.1 ± 22.0	22.1 ± 14.9	0.052

Based on data from our pilot study, the estimated effect size was 1.065. Although the pilot suggested a trend toward greater in-brace correction among patients treated with the Hybrid Bracing Protocol, the difference did not reach statistical significance, most likely due to insufficient statistical power associated with the small sample size. Recognising the exploratory nature and the methodological limitations of the pilot study, a more conservative effect size of 0.8 was therefore adopted for the purpose of sample size estimation. Assuming two-sided alpha level of 0.025 and a statistical power of 0.95, a minimum of 49 participants is required for each group. Allowing for an anticipated dropout rate of approximately 20%, the total required sample size is 120 participants, with 60 allocated to each group.

### Randomisation and blinding

Once consent has been acquired, the subjects will be randomly allocated to the conventional bracing group (CB group) or the HyBP group. Assignment to a group is by a stratified randomisation protocol with a permutated block of 10 to allow recruitment to be spread over 12 months. Allocation concealment is strictly followed to prevent investigators/subjects from knowing or predicting the next allocation. An individual sealed envelope containing the group allocation for the next subject will be open only after the subject agrees to participate and an informed consent is obtained.

### Intervention

All participants will receive the computer-aided design and computer-aided manufacture (CAD/CAM) brace from the orthotist in our institution, ideally within 6 weeks of screening to reduce the risk of curve progression before bracing, and minor adjustments will be made at subsequent follow-up to optimise the brace.

The conventional bracing group (CB group):

1. Brace fitting: The subjects in this group will be treated with the conventional rigid underarm brace (UAB) routinely used in our centre [11]. A CAD/CAM system (Vorum Research Corporation, Canada) will be used to capture the 3D information of patient’s body and to design spinal orthosis through its purpose-design software [12]. The pressure pads are strategically located based on the orthotist’s experience in examining the apex of the rib hump and lumbar prominence and information from the software. After the brace is ready, fine adjustment of location of the pressure pads will be made with reference to an in-brace whole spine radiograph.
2. Brace compliance monitor: Each brace will be fitted with a waterproof microsensor brace compliance monitor, the Orthotimer® (dimensions: 9 mm × 13 mm × 4.5 mm and weight < 1 g), from Rollerwerk-Medical engineering & consulting, Germany. The memory capacities of the brace monitor can last for 400 days. To embed the brace monitor into the brace, a dummy with the same dimensions of the monitor will be placed underneath a softened polyethylene sheet which is used to fabricate a brace. After the polyethylene becomes hard, an empty space for the monitor will be created. No extra attention is needed from the participants. Once the system is turned on, it will log the temperature at the anterolateral waist region. Data from the device will be retrieved during each study visit.

The Hybrid Bracing Protocol group (HyBP group):

1. Brace fitting: The subjects in this group will be treated with the conventional rigid underarm brace (UAB) at daytime and a nighttime lateral bending underarm brace, i.e. the lateral bending brace (LBB) [13]. The UAB will be fabricated with the same design as in the CB group. The nighttime LBB will be fabricated with the 3D information of the patient’s body as the conventional rigid underarm brace. The nighttime bending brace will be designed in a bending position opposite to the convexity of the lumbar/thoracolumbar curve. The patient will be positioned to an overcorrection posture inside the nighttime LBB. After the brace is ready, fine adjustment of location of the pressure pads will be made with reference to an in-brace whole spine radiograph. The LBB will further be adjusted until the in-brace radiograph indicates a minimum overcorrection of 10° (Cobb angle = −10°, i.e. in opposite direction to the original curve).
2. Brace compliance monitor: Both the daytime UAB and nighttime LBB will be fitted with the same waterproof microsensor brace compliance monitor as in the CB group. Data from the device will be retrieved during each study visit.

Other than treatment with different bracing protocols, the same standard orthotic and medical care for treating scoliosis will be provided for both the CB group and HyBP group according to the SOSORT “Standards of management of idiopathic scoliosis with corrective braces in everyday clinics and in clinical research” [14]. Subjects are instructed to wear the brace 23 h daily [6]. The rest hour is for bathing and exercises. Brace compliance in terms of the average number of hours per day of brace wear will be separately evaluated for the daytime UAB and nighttime LBB.

Study visits, investigations, and measurements:

1. Study visits: Subjects will be evaluated at baseline just before bracing, 3 months and 6 months after bracing, and at 6-monthly intervals thereafter.
2. Plain radiography of the whole spine in standing position will be taken with a standard protocol using the EOS system:
  1. Measurements are made as follows:
    i. PA view for measurement of:
      i. Frontal Cobb angle of the structural curve
      ii. Trunk shift will be measured in cm indicating the perpendicular distance of mid-point of T1 vertebra from the central sacral line [15].
      iii. Curve level, apex level, side of curve
    ii. Lateral view for measurement of Cobb angle for thoracic kyphosis and lumbar lordosis
3. Anthropometric and maturity assessment:
  1. Body weight, standing height, sitting height, and arm span will be measured at each study visit according to the standard protocol. The “menarchal year”, defined in a way similar to that by Lehtonen-Veromaa et al. [15], is the age at menarche subtracted from the current age (i.e. [current age − age of menarche] in years). For pre-menarchal subjects, the age of menarche is determined at subsequent study visits.
4. Brace compliance monitoring:
  1. Data on brace usage from the aforementioned monitoring devices will be retrieved at each study visit. The body temperature collected from the sensor will be used for compliance analysis [16].
5. Quality of life (QoL) questionnaires:
  1. This will be conducted according to the standard protocol at baseline and the 18-month time-point for self-administration of the following:
    1. SRS-22 [17]
    2. Spinal Appearance Questionnaire (SAQ) [18]
    3. Brace Questionnaire (BrQ) [19]
  2. The Chinese versions for SRS-22, SAQ, and BrQ have all been validated and ready for use in this study [17, 20].

### Primary outcome (in-brace correction)

i. At baseline, 3rd month, 6th month, then every 6 months thereafter
  i. PA and lateral X-ray are taken without brace for monitoring of curve severity at 3rd and 18th month.
ii. At 3rd month and 18th month
  i. PA and lateral X-ray are taken without brace, then followed by taken with the daytime UAB for evaluation of in-brace correction.
iii. At completion of brace fitting, a supine AP X-ray of the whole spine taken with LBB will be assessed with the frontal Cobb angle.
iv. In-brace Correction (IBC) = (([Pre-brace Cobb] − [In-brace Cobb])/Pre-brace Cobb) × 100%. It will be evaluated at the 3-month and 18-month time-points.
v. Radiographic measurements will be done by a single experienced rater who is blinded to subject’s grouping (he will not be shown the X-ray taken with the LBB).

### End of brace treatment

Brace treatment will be stopped with one of the following conditions:

1. At skeletal maturity: bracing will be stopped at maturity following a standardised protocol of weaning. Maturity is defined as [3]:
  1. Risser sign 4 or more and
  2. With no change in height or <1 cm change in standing height over 2 consecutive visits 6 months apart and
  3. 24 months post-menarchal
2. When surgery for scoliosis is done. The indication for surgery in our centre is Cobb angle ≥ 50° despite treatment with braces.
3. If subjects decline further treatment with bracing of their own accord

### Secondary outcome

Apart from the primary objective of assessing IBC, effectiveness of bracing treatment will also be assessed with respect to post-brace curve outcome, i.e. curve severity after cessation of bracing treatment as follows:

1. Increase in Cobb angle ≤ 5° for the structural curves at skeletal maturity.
2. Cobb angle remains ≤45° at skeletal maturity.
3. Cobb angle remains less than surgical threshold of 50° at skeletal maturity.

### Statistical methods and analysis

The spread of data will be tested for normality. For data that is normally distributed, the numerical data are expressed as mean ± SD. Otherwise they are expressed as median (interquartile range). Demographic data, anthropometric data, QoL scores, initial Cobb angle, and other curve morphological data between the two groups at baseline will be compared using two-sided independent Student’s *t*-test or chi-square test as appropriate. To analyse treatment outcomes, the “intention-to-treat” approach is adopted.

For the primary objective, two-sided independent Student’s *t*-test will be used to compare between-group IBC. For secondary objectives, chi-square test will be used for comparing the proportions of successful treatment between the two groups. Logistic regression and linear regression model will be used for control of confounding from brace compliance and variables that are unbalanced at baseline between the study groups. QoL scores at the 18-month time-point for the two study groups will be compared with ANCOVA for adjustment of effect from baseline QoL scores. A *p* value of <0.05 is considered statistically significant.

### Safety and data monitoring

The conduct, ethics, and data monitoring of the study will be overseen by our Clinical Research Ethics Committee (CREC). Potential risks of bracing include pain, impingement, and curve progression despite good compliance. The fitting of the brace will be assessed by an orthopaedic surgeon and orthotists at regular follow-up visits; the participants are advised to contact our study coordinator if they are uncertain of the discomforts. Standardisation forms will be used for monitoring and reporting adverse events. In the event of a significant adverse event, the principal investigator will report to the ethics committee within 24 h. Interim data monitoring will be performed to monitor data quality every 3 months.

### Ethics and dissemination

The study will be conducted in compliance with the Declaration of Helsinki and ICH guideline for good clinical practice (GCP). Any protocol amendments will be submitted to the ethics committee for approval as required. The patients and public were not involved in the design, conduct, or reporting of our research. The findings of this study will be disseminated through regional and international meetings and through peer-reviewed scientific articles. The de-identified data can be obtained from the corresponding author upon reasonable request.

## Trial status

The current version of the study protocol is V.02, dated 12 March 2021. Participant recruitment into the RCT was delayed due to the COVID-19 pandemic. The first participant was enrolled on 20 Oct 2021, and the final participant was enrolled on 6 May 2024. Recruitment is now complete. This protocol manuscript was submitted after completion of participant recruitment; however, outcome data collection and follow-up were ongoing at the time of submission, and no analyses had been conducted.

Statistical analysis of the primary and secondary outcomes for this phase 1 RCT will be performed after the final recruited participants have reached 18 months of bracing, with the final 18-month assessment expected in July 2026. The final analysis of bracing success will be conducted once all participants have reached skeletal maturity. Completion of bracing for all participants, which is dependent on attainment of skeletal maturity, is anticipated in 2027, with 2-year post-treatment follow-up expected to be completed in 2029.

## Conclusion

The results of this hybrid brace study will enhance an evidenced-based understanding of combining the advantages of underarm brace with a nighttime overbending brace for optimising in-brace correction, and eventually effectiveness in preventing curve progression and the need for major spinal surgery.

## Supplementary Information

Supplementary Material 1.

Supplementary Material 2.

## Data Availability

De-identified data from this study will be available from the corresponding author upon reasonable request.
